# Observed communication skills: how do they relate to the consultation content? A nation-wide study of graduate medical students seeing a standardized patient for a first-time consultation in a general practice setting

**DOI:** 10.1186/1472-6920-7-43

**Published:** 2007-11-08

**Authors:** Tore Gude, Per Vaglum, Tor Anvik, Anders Baerheim, Hilde Eide, Ole B Fasmer, Peter Graugaard, Hilde Grimstad, Per Hjortdahl, Are Holen, Tone Nordoy, Helge Skirbekk, Arnstein Finset

**Affiliations:** 1 Department of Behavioral Sciences in Medicine and Institute of General Practice and Community Medicine, Faculty of Medicine, University of Oslo, Norway; 2 Institute of Community Medicine and Institute of Clinical Medicine, Faculty of Medicine, University of Tromsø, Norway; 3 Department of Public Health and Primary Health Care and Institute of Clinical Medicine, Faculty of Medicine, University of Bergen, Norway; 4 Department of Public Health and General Practice, Department of Neuroscience, Faculty of Medicine, Norwegian University of Science and Technology, Trondheim, Norway; 5 Faculty of Nursing, Oslo University College, Norway

## Abstract

**Background:**

In this study, we wanted to investigate the relationship between background variables, communication skills, and the bio-psychosocial content of a medical consultation in a general practice setting with a standardized patient.

**Methods:**

Final-year medical school students (N = 111) carried out a consultation with an actor playing the role of a patient with a specific somatic complaint, psychosocial stressors, and concerns about cancer. Based on videotapes, communication skills and consultation content were scored separately.

**Results:**

The mean level of overall communication skills had a significant impact upon the counts of psychosocial issues, the patient's concerns about cancer, and the information and planning parts of the consultation content being addressed. Gender and age had no influence upon the relationship between communication skills and consultation content.

**Conclusion:**

Communication skills seem to be important for final-year students' competence in addressing sensitive psychosocial issues and patients' concerns as well as informing and planning with patients being representative for a fairly complex case in general practice. This result should be considered in the design and incorporation of communication skills training as part of the curriculum of medical schools.

## Background

Effective patient care relies on the physician's understanding of the patient's biological, psychosocial, and cultural background. In the initial consultation with a patient in a general practice setting, the optimal content of a consultation constitutes the basis for the following: reliably diagnosing biological as well as relevant psychosocial factors, exploring the patient's ideas, concerns, and expectations, and informing the patient about his or her condition including a plan for further treatment [1,2].

The training in specific communication skills during medical school is based on the notion that independent of the physician's medical knowledge, the practice of communication skills will have a significant impact upon the character and quality of the consultation. The reason for consulting the doctor may be concerns about more than symptom-related distress [3]. In some studies, successful levels of communication skills have been linked to students' diagnostic efficiency, patient stress, patient compliance, medical errors, general outcome, outcome in chronic disorders, and patient and physician satisfaction [3-12]. However, the intermediate variable between communication skills and such outcome measures, the content of a consultation in a general practice setting, has still not been sufficiently explored.

Our hypothesis has been that there is a relatively strong association between communication skills and the content of the consultation. We are well aware of the claims that focus on training in communication skills may be at the expense of basic doctoring skills. Further, if adequate somatic knowledge exists, communication skills may have only a marginal additional impact upon the content of the consultation [13].

One way to explore these issues is to observe students or physicians in their interaction with patients in a clinical setting. In this regard, one basic methodological problem should be addressed. Should one employ a standardized patient (i.e. an actor in a constructed but typical patient setting) or many different patients? This study design has adopted the first option so all interviewers attending the study could be compared in their skills, but the generalization of the findings may be limited to the specific case being played by the actor, even if the case in its relative complexity is fairly representative of patients in general practice. Findings indicate that a significant case-by-student interaction between communication skills and decision making exists and that individuals' communication skills vary systematically with specific cases [14]. The length of the test being observed may also influence the results. A 15-minute consultation, ordinary in a general practice setting and part of the present study design, has been found to be too short to assess generalizable interviewing skills [15]. Concerning background variables, we would presume that female and older students will develop communication skills promoting psychosocial issues more easily and more effectively than males and younger students.

We have conducted an observational study of a consultation with a standardized and common primary care patient performed by graduate medical students from all universities in Norway. Our research questions were:

1. Will overall level of communication skills be related to the content part like the somatic, the psychosocial, the cancer concern, and the informing/planning indices addressed in the consultation when controlled for gender and age?

2. Will certain characteristics like gender, age, and level of communication skills differ between students who obtain high scores on the somatic, the psychosocial, and the information/planning indices of the consultation from those obtaining low counts?

## Methods

### Subjects

Final-year medical students (N = 320) attending all four medical schools in Norway in the spring of 2004 were invited to participate in a test examination about two months before their graduation. The aim was to recruit 30 students from each site. At two schools, this number of students responded to the first call by mail. At the third school, 23 students initially responded, and five more responded after the first reminder, raising the number to 28. At the fourth school, only 12 students initially responded; after two reminders, the number increased to 23. Thus, the sample to be investigated consisted of 111 students, 70% women (N = 78), aged 28 ± 3 (range 23 – 45).

The sample has to be viewed as an "availability" sample, and we cannot know for sure how representative the attendees were of the national student population. The data from our survey sample showed that the gender proportion among the final-year students in spring 2004 (N = 320) was 65% females (N = 208, ratio 0.65, CI 95% 0.60 – 0.70); in the observational sample (N = 111 of the same 320), the gender proportion of females was 70% (N = 78, ratio 0.70, CI 95% 0.61 – 0.78). Mean age among the 320 was 27,3 (CI 95% 26,9 – 27,7) years, among the 111 27,8 (CI 95% 27,2 – 28,4). When we compared the scores on a self-report questionnaire assessing their *self evaluation *of communication skills (Oslo Inventory of Self-reported Communication Skills – OSISCS), the participants in the survey study yielded approximately the same mean score (3,75 +0.46 on a 1–5 scale) one year earlier (when they were in their 5^th ^year of medical school; medical school in Norway lasts for six years) as the 111 attendees (3,51 +0.34) with minor variance between genders [16]. With overlapping confidence intervals regarding gender and age and close to similar scores on self-reported skills, none of these differences should be viewed as significant. Thus we presume that we have achieved a fairly representative sample.

### Medical schools

The four medical schools in Norway use identical governmental admission criteria, have the same curriculum length (six years), but have some differences in design. One of the schools offers a "traditional" curriculum divided into a pre-clinical and a clinical part. The remaining three schools do not have this sub-division, running "integrated and/or problem-based" curricula, where students see patients from the onset. Since the differences between schools are not extensive and given the limited sample size (N = 111), we have run the analyses without differentiating between curricula.

### The standardized patient

A common primary patient's clinical story with multiple problems was constructed through discussions within the research team comprising nine experienced clinicians (four GP's, one oncologist and four psychiatrists). The patient was a 43-year-old woman visiting this "physician" for the first time, complaining of irregular menstrual bleedings (see the case story in the Appendix). Her story also contained psychosocial distress related to recent of divorce and relocation, a stressful job and a fear of uterine cancer, of which her mother may have died 10 years earlier. During the consultation, she was to appear as an avoiding and non-complaining person, not disclosing her concerns easily.

Four professional actors, one from each of the four university cities, were instructed to simulate this patient. A professional instructor was hired to train them together in order to standardise the role as much as possible.

### The consultation

The consultations were carried out at each of the four schools and all interviews were videotaped. Prior to the consultation, the students completed the OSISCS and received the same instructions: They were told that they were in their obligate part of the postgraduate period working as an assistant with a general practitioner and had 15 minutes available to see a female patient, NN, 43 years old, for her first consultation due to irregular menstrual bleeding. Their task was to carry out a complete first-time consultation in a general practice setting. They might propose examinations/laboratory tests to be undertaken during the actual visit, getting the results then and there. Further examinations had to be planned as part of the follow-up procedure (see Appendix).

### Assessment of the consultation content – outcome variables

A rating instrument, Consultation Content, with 15 dichotomous items related to consultation content for this particular patient, was constructed after thorough discussions within the research group (Table 1). Two independent raters scored the videotapes according to this list of consultation content items as zero (not present) or 1 (present) according to whether the student-physician had addressed the specific issue or not in the consultation. Thus, total content counts could range between zero and 15.

**Table 1:** Items used in evaluating the consultation content.

Has the student performed/addressed:		
A. Biomedical diagnoses:		
1. Myomas (as cause of the irregular bleedings)	□ yes	□ no
2. Low hemoglobin	□ yes	□ no

B. Subjective complaints		
3. Exhaustion	□ yes	□ no
4. Headache	□ yes	□ no
5. Vertigo	□ yes	□ no
6. Sleep problems	□ yes	□ no
7. Reduced appetite	□ yes	□ no

C. Concern aspects		
8. Mother dead from uterine cancer	□ yes	□ no
9. Fear of inherited cancer	□ yes	□ no

D. Psychosocial aspects		
10. Three children	□ yes	□ no
11. All responsibility for them	□ yes	□ no
12. Divorced one year ago	□ yes	□ no
13. Moved to a new place during the last year	□ yes	□ no
14. Exploring the job situation	□ yes	□ no
15. Problems with divorce	□ yes	□ no

E. Informing/planning:		
Treatment plan (MAAS – 6)	□ yes	□ no
Information (MAAS – 9)	□ yes	□ no

The two raters obtained very similar counts and a high correlation (r = 0.85) between them.

Since the construction of the content scale was not based upon psychometric properties, we found it most reasonable to look at the face validity. The 15 consultation content items (Table 1) were divided into three Content indices: The Somatic Index comprising the two diagnostic items (myomas and anemia) together with the five illness items (numbers 3 – 7). Due to very low internal consistency (Cronbach's α = 0.26), the two diagnostic items were excluded and with the five illness items left constituting the Illness index (1), alpha increased to 0.44. The Concern Index (2) comprised two items related to the patient's concern about possible cancer (numbers 8 – 9, α = 0.53), and the Psychosocial Index (3) comprised the six psychosocial items (numbers 10 – 15, α = 0.46).

We also constructed a fourth index called The Informing/planning Index (4) by combining two items (item 6 – treatment planning and item 9 – information, α = 0.72) from the Maastricht History and Advice Checklist (MAAS) [17]. This index covered the action taken by the student at the end of the consultation. MAAS items were scored on a 0–5 scale, yielding a median of 3,0. A cut off >3 was used to demonstrate presence of the item during the consultation. Inter-rater reliability between the MAAS raters was (ICC(1.1)) = 0.69.

We then divided the students into groups with high or low counts on the Content indices by merging the Psychosocial and Concern indices into a Psychosocial/concern index in order to simplify the classification. Together with the Illness and the Informing/planning indices, these three indices were dichotomized at their medians. By combining high and low scores on the indices, eight sub-groups emerged with different combinations (from all high to all low). These groups would then be tested against level of communication skills in order to check for correlation linearity.

### Assessment of communication skills

The Arizona Communication Interview Rating Scale (ACIR) consists of 14 items (Table 2) all with a scale from 1 (least) to 5 (best) [18]. Three trained raters scored the videotapes and one separate rater scored 2/3 of them in order to check the inter-rater reliability (not the same as above). The Intra-class correlation coefficient between raters was similar to that between the MAAS raters ICC(1.1) = 0.69. The overall ACIR mean was 3,10 (0.69). The psychometric properties of ACIR have been found satisfactory in an earlier study [19]. A Principal Component Analysis with a Varimax rotation yielded a typical one-factor solution, so the overall mean was used in all analyses. The internal consistency of ACIR in our sample was 0.91 (Cronbach's α). As one way of validating ACIR, we correlated ACIR-mean against the MAAS-mean (without the two items constituting the Informing/planning index being used as a content index) giving a value of Pearson's r = 0.61.

**Table 2:** Item-list for Arizona Communication Interview Rating Scale (ACIR)

	1	2	3	4	5
Items	(lowest score)			(highest score)	
1. Organization	□	□	□	□	□
2. Timeline	□	□	□	□	□
3. Transitional utterances	□	□	□	□	□
4. Open questioning	□	□	□	□	□
5. Smooth progress	□	□	□	□	□
6. Avoiding repetition	□	□	□	□	□
7. Summarizing	□	□	□	□	□
8. Understandable information	□	□	□	□	□
9. Documentation	□	□	□	□	□
10. Eye contact	□	□	□	□	□
11. Attentiveness	□	□	□	□	□
12. Response to concerns	□	□	□	□	□
13. Feed-back	□	□	□	□	□
14. Additional questions	□	□	□	□	□

The question of tautology may be raised as the rating of the ACIR items could be influenced by how the content items were rated and vice versa, even if separate raters were used. We, therefore, wished to control for this possibility using a special procedure. When carefully scrutinizing the ACIR items one by one, we categorized them as content-related and non-content-related. Items number 3, 4, 5, 6, 8, 10 and 11 were considered non-content-related. This categorization, which of course may be disputed, is highly consonant with the separation of ACIR items performed in a previous study by Aspegren et al. [20]. This group of items (numbers 5, 6, 8, 10, and 11, Cronbach's α = 0.86) was viewed as more typical for civil social conversation, i.e. not strictly related to a specific medical context. The mean of these non-content related items could be used as a substitute for the ordinary overall ACIR-mean in the multiple analyses to test for possible differences in association with our outcome variables (Content indices).

### Statistics

Means, frequencies, Anova, Linear Regression, Principal Component Analysis with Varimax rotation, and Reliability testing were conducted in the SPSS 13.0 version.

## Results

The frequency of every single content item being addressed in the consultation is shown in Table 3, varying from 95% of the consultations concerning exhaustion (Somatic) to 10 % concerning divorce problems (Psychosocial). The content of Myomas and anemia was addressed in 57% and 43% respectively. Descriptive data concerning overall ACIR mean and total counts of Content indices present in the consultation by gender and age groups are shown in Table 4. No significant differences were detected between female and male students or age groups.

**Table 3:** Frequency of the 15 consultation content items addressed during the interview.

Consultation content items (numbers from Table 2)	How frequently addressed %
Exhaustion (3)	95,5
Three children (10)	87,4
Mother dead of ca. (8)	64,9
Headache (4)	64,4
Divorce (12)	58,6
Concern for own ca. (9)	57,7
Myomas (1)	56,8
Anemia (2)	43,2
Insomnia (6)	42,3
Job situation (14)	34,2
Moved (13)	32,4
Vertigo (5)	29,7
Responsibility (11)	27,9
Appetite (7)	27,0
Divorce problems (15)	9,9

**Table 4:** Levels of ACIR overall mean and consultation content total count by gender and age groups.

	ACIR overall mean	Anova F-value	Consultation content count	Anova F-value
Whole sample	3,10 (0.69)		8,42 (2,35)	
Female	3,09 (0.70)	0.02 n.s.	8,46 (2,40)	0.07 n.s.
Male	3,11 (0.70)		8,33 (2,26)	
Age < 26 yrs.	2,92 (0.63)	0.48 n.s.	8,08 (2,22)	0.17 n.s.
Age 26–30 yrs.	3,12 (0.68)		8,48 (2,92)	
Age > 30 yrs	3,13 (0.91)		8,36 (3,07)	

In four linear regression analyses, the four consultation Content indices (Illness, Psychosocial, Concern, and Informing/planning) were used as dependent variables and the overall ACIR-mean as the independent variable with gender and age as control variables. The level of communication skills had a significant impact upon the Psychosocial, the Concern and the Informing/planning indices, controlled for gender and age, yielding standardized Beta-values of 0.45 for the concern index (explaining 19% of the variance), .34 for the Informing/planning index (11%), and 0.29 for the psychosocial index (8%). The Illness index had no significant relationship to level of communication skills (Table 5). When substituting the overall ACIR-mean with the ACIR non-content-related mean, no deviating associations were detected (Beta-values deviating at a maximum of 0.02).

**Table 5:** Standardized Beta-values from Linear Regression Analyses for Arizona overall mean as predictor for the four consultation content indices controlled for gender and age.

	Standardized Beta-values			
	Illness	Psycho-social	Concern	Inform/plan
Gender	0.06	-0.15	0.01	0.01
Age	-0.04	0.02	0.03	0.09
ACIR	-0.11	0.29**	0.45***	0.34**
Expl.var. R^2^	0.04	0.08	0.19	0.11

** p < 0.01, *** p < 0.001, no significance = n.s.

When exploring characteristics for the eight sub-groups derived from combinations of the dichotomized Illness, Psychological/concerns, and the Informing/planning part-counts, overall ACIR-mean was significantly higher in those with high score (above median) on all three indices (Illness, Psychosocial/concern, and Informing/planning) compared with those having low score on all three or high on the Illness but low on the two others (F = 4,60, p = 0.001). Gender and age did not vary between the eight sub-groups.

## Discussion

One of the main findings in this study is that the level of communication skills was significantly related to the total content index, especially to the Psychosocial, the Concerns, and the Informing/planning indices, but not to the Illness index, controlled for gender and age. As shown in Table 5, the strongest relationship was with the Concern index, while the Informing/planning and Psychosocial indices yielded somewhat weaker relationships. This implies that the higher the level of communication skills, the more adequately addressed was the content of the consultation related to the patient's possible stressors; i.e. her fear of having the cancer from which her mother may have died 10 years earlier, responsibility for her children, the divorce she had been through, and the burden of moving to a new place, a.o. The students with the higher level of communication skills were also more able to perform adequate informing/planning procedures at the end of the consultation.

These results were supported by the fact that students scoring high on all three dichotomized indices (the Illness, the Psychosocial/Concerns, and Informing/planning) had significantly higher levels of communication skills than those scoring low on all three indices, as well as those with high score on the Illness, but low scores on the other two indices. No variation in gender and age characterized these sub groups, showing that it was the level of communication skills that mattered. The risk of tautology when exploring associations between phenomena with instruments possibly covering some of the same issues has been considered and attended to by testing any possible difference when using the overall mean in contrast to the no-content-related mean of ACIR. When no such difference existed, it is reasonable to presume that the relationship we have found between communication skills and the content indices in this specific consultation was real and not confounded by lack of construct validity for the two instruments.

The finding that there was no significant relationship between a high level of communication skills and a high count on the Illness index is important. This may be due to a low variance in the Illness index, but this does not coincide with Table 3. Another interpretation of this finding, therefore, may be that the somatic content of this particular consultation (the diagnoses of possible myomas and anemia as well as the subjective complaints) depends more on biomedical knowledge than the communication skills themselves and that the presence of these symptoms was not related to psychosocial aspects. Along the same line, we found that those who scored high on the Illness index but low on the Psychosocial and Informing/planning indices had a significantly lower level of communication skills compared with those with high scores on all three indices. This supports the view that graduate students who are very competent in evaluating somatic complaints nevertheless may fail to identify important psychosocial stressors and concerns, in this case due to the lack of communication skills. We know that these issues are important both for patient compliance and satisfaction as well as for the further course and outcome of many somatic disorders [6,8-12]. In order to obtain valuable information on the emotional burdens of patients who may be less willing than the "patient" in this study to disclose such issues, the need for specific communication skills is obvious. In a study by Pfeiffer et al. [21], a decrease in students' communication skills was found through the last part of medical school, after an initial increase, especially their skills in obtaining an adequate social history. One interpretation offered was that this can be due to a medical culture which de-emphasizes communication skills in favor of somatic knowledge. This can also be a possible explanation of our findings. Therefore it is important to identify factors that may enhance as well as hamper the development of communication skills among medical students.

This study has strengths and limitations. The strength is the nation-wide sample, while a limitation can be the representativeness of our sample (N = 111) compared with the available cohort of last year's students (N = 320). We cannot overlook the possibility that the students who participated in our study were more motivated than those who did not attend. Such motivation could be based upon an understanding of the need to focus upon communication skills or it could be the wish to test their level of skills shortly before their final exams. With no deviance in gender proportion and mean age, nor in the level of self-assessment of communication skills that the 320 students reported in their fifth year compared to what the 111 out of the same 320 students reported one year later, we may presume that the representativeness may be acceptable.

Another limitation is of course the single patient case used in this study. Even if we may argue that our case story is representative for a patient consulting a general practitioner, we should be cautious in generalizing our conclusions regarding the students' communication skills and the relation to consultation content too far. As mentioned above, previous studies have shown the need for more comprehensive studies with observation of communication skills based on more variation in cases than we have used in this study [14,15].

Another limitation could be the variation in the actors' performances of the patient role. Even if they were instructed and trained together preceding the interviews, some variation in their withdrawal behaviour could be observed in the videotapes.

According to the instructions the students were given before interviewing, max. 15 minutes should be used for interviewing. A few of them used up to 18 minutes, but as the counts did not improve with more time, this factor was not considered important.

## Conclusion

This study has shown that the level of communication skills and the content of the consultation with regard to psychosocial issues, patient concerns and the informing and planning procedures (with a representative patient in a general practice setting) among graduate medical students are significantly correlated. On the other hand, no such relationship between communication skills and the somatic/illness content of the consultation was detected. Even if the students attending this study had good biomedical knowledge on this specific case, they may have overlooked important psychosocial stressors as well as the patient's concerns, and may have lacked the necessary skills to conclude this single patient consultation in a fruitful way. In this light, medical schools and researchers, utilizing a broader spectrum of patient stories, should continue to identify factors that promote as well as inhibit medical students' learning of communication skills.

## Competing interests

The author(s) declare that they have no competing interests.

## Authors' contributions

TG participated in the design of the study, organized and took part in the data collection, scored the interviews, performed the data analyses and were the main writer, PV participated in the design of the study and gave major contributions to the writing, TA participated in the design of the study, took part in the data collection, and contributed to the writing, AB participated in the design of the study, took part in the data collection, and contributed to the writing, OBF participated in the design of the study, took part in the data collection, and contributed to the writing, HE scored the interviews and contributed to the writing, PG scored the interviews and contributed to the writing, HG participated in the design of the study, took part in the data collection, and contributed to the writing, PH participated in the design of the study, took part in the data collection, and contributed to the writing, AH participated in the design of the study, took part in the data collection, and contributed to the writing, TN participated in the design of the study, took part in the data collection, and contributed to the writing, HS scored the interviews and contributed to the writing, and AF scored the interviews and contributed to the writing.

## Appendix

Instructions to the actor

- You are going to play the role of Eva, a 42 year old woman, divorced and working at the local library. You have an avoidant personality structure and you look pale and exhausted when you enter the office, sitting down in the chair you are offered with a heavy sigh as it is good to be seated.

The most typical characteristics of avoidance are:

- A pervasive pattern of social inhibition

- Feelings of inadequacy

- Hypersensitivity to negative evaluations

- Avoiding working situations involving cooperation with others due to fear of critics

- Restraint within intimate situations due to fear of being shamed or ridiculed

- Views self as socially inept, personally unappealing, or inferior to others

### Complaints – Illness

To the doctor's initial question, you tell that the reason for coming is irregular menstrual bleedings. When the doctor asks you to tell more about it, you start with telling about a duration of half a year with irregular intervals and stronger, more lasting bleedings than before. On this background you asked for a consultation, and got one in two weeks.

If the doctor asks you about your own opinion of the condition, you may disclose your feeling of exhaustion and thoughts about an approaching menopause. But you have been increasingly worried about something serious turning up. Up till the last year, you have had regular menstrual bleedings with 29 days between each starting point.

But the last half of a year, bleedings have appeared with form 10 to 34 days intervals, lasted for eight to nine days (earlier five to six days). Now you have to use double menstrual pads to avoid bleeding through, but yesterday it happened and last night you had to rise from bed in order to change pads due to fresh red blood with cloths appearing.

Your first menstrual bleeding occurred when you were 12 years old. You have had four pregnancies, given birth to three children. The fourth pregnancy terminated in a spontaneous abortion two years after your second child was born.15 years ago. All labours were uncomplicated. After the divorce one year ago, you have not established any new intimate relationship due to fear of one more failure. Therefore, you have stopped using prevention.

You smoke 10 cigarettes a day and use no medication except some occasional Aspirin due to headache with some relief.

You have up to now had a good health with no need for consulting a doctor after you moved to this new place, where you now live, one year ago.

If and when the doctor asks whether you need a sick-leave, you respond that it could be good for you, but you are not sure if you are ill enough and therefore have to give a self-report instead.

If the doctor asks more about your life situation, you may tell (not necessarily disclose all of it immediately) that you have moved from your earlier living place into a new flat as the only provider for your three children because of the divorce from your husband one year ago. Your economy is strict as you have to pay high interest on your loan, but you are in balance.

Your three children are: Nils 18 years (high school), Trine 15 years (junior high school) and Peter 5 years (day-care centre). Your ex-husband, working on an oil-drilling platform met another woman and moved together with her far away.

You have no contact with him, he calls once in a while the children. They have adapted pretty well to the new surroundings. You have not been able to establish a social network in your new living place, are afraid of taking initiative as others may think you are silly and uninteresting to join with.

You are educated as a librarian and got the job with only a 15 min. walk from home.

In the beginning you liked it, but he last months you have dreaded going to work feeling insufficient. Your new boss demands everyone to be creative and starting new projects thus, favouring your peers doing this, while the more withdrawn, like yourself, become scapegoats.

You try to avoid conflicts with your boss, but she is more and more critical towards you. If the doctor asks you explicitly about your real situation, you disclose your feeling of increasing bad temper, exhaustion, and problems with insomnia leading to tiredness in the morning. Your appetite is reduced, but you have not lost weight.

If depressions in the family, especially mother, are explored by the doctor, you answer "not as far as I know".

The last couple of months you have had some headache, had vertigo and had problems with reading as the letters have merged together.

You have less energy to mobilize and have thought a lot about your mother who died only 52 years old from "cancer in her belly" some 10 years ago.

With hesitation, you disclose fear of the same faith hitting you after you have got these menstrual problems. Uterine cervix smear two years ago was normal.

If you have the opportunity you may disclose your concern about reduced ability to care for your children. Your two teen-agers are in opposition to and criticize you. You feel it as a burden when they quarrel a lot and it is difficult for you to intervene in their expanding activities. It is a lot more drugs and alcohol abuse among young people here than where you came from and you feel uncomfortable with Trine's dating a 10 year older truck driver.

Results from the investigation (to be informed about on the student's request):

At the gynaecological investigation, abundant blood with some cloths occurs. The uterine body is enlarged as with eight weeks pregnancy. The consistency is flexible with a bulky surface. Both ovarian are normal. Blood pressure 135/80, hearth rate 72, regular, Hb.10.5. Additional clinical investigations are normal.

## Pre-publication history

The pre-publication history for this paper can be accessed here:



## References

[B1] Kurtz S, J S, J D (2005). Teaching and learning communication skills in medicine.

[B2] Pendleton D (2003). The New consultation: developing doctor-patient communication.

[B3] Smith RC, Greenbaum DS, Vancouver JB, Henry RC, Reinhart MA, Greenbaum RB, Dean HA, Mayle JE (1990). Psychosocial factors are associated with health care seeking rather than diagnosis in irritable bowel syndrome. Gastroenterology.

[B4] Little P, Everitt H, Williamson I, Warner G, Moore M, Gould C, Ferrier K, Payne S (2001). Observational study of effect of patient centredness and positive approach on outcomes of general practice consultations. BMJ.

[B5] Roter DL, Hall JA, Katz NR (1987). Relations between physicians' behaviors and analogue patients' satisfaction, recall, and impressions. Med Care.

[B6] Mead N, Bower P (2002). Patient-centred consultations and outcomes in primary care: a review of the literature. Patient Educ Couns.

[B7] Anderson LA, Zimmerman M (1993). Patient and Physician Perceptions of their Relationship and Patient Satisfaction: A Study of Chronic Disease Management. Patient Educ Couns.

[B8] Evans BJ, Stanley RO, Mestrovic R, Rose L (1991). Effects of communication skills training on students' diagnostic efficiency. Med Educ.

[B9] Roter DL, Hall JA, Kern DE, Barker LR, Cole KA, Roca RP (1995). Improving physicians' interviewing skills and reducing patients' emotional distress. A randomized clinical trial. Arch Intern Med.

[B10] DiMatteo MR, Sherbourne CD, Hays RD, Ordway L, Kravitz RL, McGlynn EA, Kaplan S, Rogers WH (1993). Physicians' characteristics influence patients' adherence to medical treatment: results from the Medical Outcomes Study. Health Psychol.

[B11] Kempainen RR, Migeon MB, Wolf FM (2003). Understanding our mistakes: a primer on errors in clinical reasoning. Med Teach.

[B12] Kaplan SH, Greenfield S, Ware JE (1989). Assessing the effects of physician-patient interactions on the outcomes of chronic disease. Med Care.

[B13] Wall D, Bolshaw A, Carolan J (2006). From undergraduate medical education to pre-registration house officer year: how prepared are students?. Med Teach.

[B14] Guiton G, Hodgson CS, Delandshere G, Wilkerson L (2004). Communication Skills in Standardized-Patient Assessment of Final-Year Medical Students: A Psychometric Study. Advances in Health Sciences Education.

[B15] Stillman P, Swanson D, Regan MB, Philbin MM, Nelson V, Ebert T, Ley B, Parrino T, Shorey J, Stillman A, . (1991). Assessment of clinical skills of residents utilizing standardized patients. A follow-up study and recommendations for application. Ann Intern Med.

[B16] Gude T, Baerheim A, Holen A, Anvik T, Finset A, Grimstad H, Hjortdahl P, Risberg T, Vaglum P (2005). Comparing self-reported communication skills of medical students in traditional and integrated curricula: A nationwide study. Patient Educ Couns.

[B17] van Thiel J, Kraan HF, van der Vleuten CP (1991). Reliability and feasibility of measuring medical interviewing skills: the revised Maastricht History-Taking and Advice Checklist. Med Educ.

[B18] Stillman PL (1980). Arizona Clinical Interview Medical Rating Scale. Med Teach.

[B19] HF K, Crinjen AAM, Vleuten, T. I, Jr. LL, Putnam SM and A.Lazare  (1995). Evaluating instruments for medical interviewing skills. The Medical Interview Clinical Care, Education and Research.

[B20] Aspegren K, Lonberg-Madsen P (2005). Which basic communication skills in medicine are learnt spontaneously and which need to be taught and trained?. Med Teach.

[B21] Pfeiffer C, Madray H, Ardolino A, Willms J (1998). The rise and fall of students' skill in obtaining a medical history. Med Educ.

